# DCFF-MTAD: A Multivariate Time-Series Anomaly Detection Model Based on Dual-Channel Feature Fusion

**DOI:** 10.3390/s23083910

**Published:** 2023-04-12

**Authors:** Zheng Xu, Yumeng Yang, Xinwen Gao, Min Hu

**Affiliations:** 1 SHU-SUCG Research Centre of Building Information, Shanghai University, Shanghai 201400, China; 2 SILC Business School, Shanghai University, Shanghai 201800, China; 3 School of Mechatronic Engineering and Automation, Shanghai University, Shanghai 200444, China

**Keywords:** multivariate time-series, anomaly detection, short-time Fourier transform

## Abstract

The detection of anomalies in multivariate time-series data is becoming increasingly important in the automated and continuous monitoring of complex systems and devices due to the rapid increase in data volume and dimension. To address this challenge, we present a multivariate time-series anomaly detection model based on a dual-channel feature extraction module. The module focuses on the spatial and time features of the multivariate data using spatial short-time Fourier transform (STFT) and a graph attention network, respectively. The two features are then fused to significantly improve the model’s anomaly detection performance. In addition, the model incorporates the Huber loss function to enhance its robustness. A comparative study of the proposed model with existing state-of-the-art ones was presented to prove the effectiveness of the proposed model on three public datasets. Furthermore, by using in shield tunneling applications, we verify the effectiveness and practicality of the model.

## 1. Introduction

In real-world applications, complex systems and devices, such as smart grids, water treatment systems, shield machines, and self-driving cars, typically contain multiple sensors. During operation, these sensors generate large quantities of time-series data that are often interrelated. Abnormal changes in the data from one sensor can affect the data from other sensors, making it increasingly difficult to detect anomalies in this growing volume and dimension of data. The development of efficient and accurate multivariate time-series anomaly detection algorithms is crucial for the continuous monitoring of key indicators or parameters in these systems and devices, ultimately increasing their level of automation.

In recent years, deep learning has become a widely adopted tool for time-series data analysis [1], with the ability to effectively extract both temporal and spatial features. At present, multivariate time series anomaly detection is mainly divided into the following three methods: (1) Temporal correlation-based method, where each variable is impacted by historical data values. Recurrent neural networks (RNNs) [2,3,4,5,6] and temporal convolutional networks (TCNs) [7] have gained popularity for their ability to extract temporal features. RNNs store past information in time and automatically extract advanced features from historical data, while TCNs provide greater flexibility in changing the size of the receptive field, enabling better control over the memory length of the model. (2) Spatial correlation-based method, which describes the correlation between different variables. In terms of extracting spatial features, graph neural networks (GNNs) and their variants [8,9,10] have played a critical role. GNNs view each variable in multivariate data as a node and the relationships between variables as edges. This allows the GNN to learn the relationships between variables and extract potential spatial features. However, with an increasing number of sensors, the use of GNNs can lead to higher space complexity and greater memory requirements when learning these relationships. (3) The method for spatial-temporal correlation fusion, of which Transformer is a typical model [11,12,13]. Transformer’s self-attention mechanism can capture the potential correlation between sequences, and its position encoding and upsampling algorithm can capture multi-scale temporal information. However, it is worth noting that the training process of the transformer-based model requires powerful computer hardware and the complexity of the model may hinder its deployment in practical engineering projects.

Recurrent neural networks are prone to gradient disappearance or gradient explosion when processing large amounts of data. The greater the number of sensors, the greater the memory occupied by the graph neural network in extracting spatial features from multivariate data. The model based on transformer has high requirements on equipment hardware in the training stage, coupled with the harsh site environment of shield engineering projects, so it is difficult to apply to shield engineering. Therefore, we propose a stable and practical model for multivariate time-series anomaly detection. The model is based on a dual-channel feature extraction module. Unlike previous studies in the literature, our model combines spatial STFT to extract spatial features and the time-based graph attention layer to extract temporal features. These features are then fused and fed into the subsequent network structure. As a result of the fusion of different feature information, the proposed method can be more accurate and robust in its detection of anomalies.

The contributions of the paper are as follows:

1. A multivariate time-series anomaly detection model based on dual-channel feature fusion (DCFF-MTAD) is proposed.
2. A spatial short-time Fourier transform module is presented for fully extracting spatial features from multivariate data.
3. In order to improve the robustness of the anomaly detection model, the Huber loss is introduced.
4. Our network shows good performance on three publicly available datasets.
5. Extensive ablation experiments are conducted to investigate the key factors improving anomaly detection performance.

The structure of this paper is organized as follows. In Section 2, we present the literature related to deep learning methods and short-time Fourier transform in anomaly detection of multivariate time-series. Section 3 shows the dual channel feature extraction module, feature fusion module, and anomaly detection module. In Section 4, we conducted the comparative experiment with existing state-of-the-art models and the ablation study of our model. In Section 5, we verify the effectiveness and practicability of our model in shield tunneling engineering. In Section 6, we present the conclusion and the prospect of future research work.

## 2. Related Work

In this section, we first review deep learning method in multivariate time-series anomaly detection, and since our model relies on the short-time Fourier transform, we also summarize related research works on the topic.

### 2.1. Deep Learning Method

Since data in complex systems or devices often lack anomaly labels, the problem of anomaly detection is usually regarded as an unsupervised learning problem. During the past few years, researchers have proposed many effective methods for unsupervised anomaly detection.

#### 2.1.1. Temporal Correlation-Based Method

Zong et al. [14] proposed a deep autoencoder Gaussian mixture model (DAGMM) for unsupervised anomaly detection, which utilizes a deep autoencoder to generate a low-dimensional representation and reconstruction error for each input data point, and further fed it into a Gaussian mixture model. DAGMM jointly optimizes the parameters of the deep autoencoder and the hybrid model in an end-to-end manner and utilizes a separate estimation network to facilitate the parameter learning of the hybrid model. Hundman et al. [15] used LSTM for anomaly detection in spacecraft telemetry systems and proposed a new non-parametric dynamic threshold (NDT) method that does not rely on scarce labels or spurious parametric assumptions. Li et al. [16] proposed an unsupervised multivariate anomaly detection method based on generative adversarial network (GAN), which uses long short-term memory recurrent neural network (LSTM-RNN) as the basic model in the GAN framework (i.e., generator and discriminator) to capture the temporal correlation of the time-series distribution. Instead of processing each data stream independently, the method considers the entire set of variables simultaneously to capture potential interactions between variables. Audibert et al. [17] proposed USAD, an autoencoder-based approach for unsupervised anomaly detection in multivariate time-series, and conducted adversarial training inspired by generative adversarial networks. Its autoencoder structure makes it an unsupervised method and enables it to show great stability in adversarial training. Su et al. [18] proposed OmniAnomaly, which uses key technologies such as random variable connection and plane normalization flow to learn a robust representation of multivariate time-series, obtain its normal pattern, reconstruct input data according to these representations, and detect anomalies using reconstruction probability. Abdulaal et al. [19] extracted the priors of multivariate signals through spectrum analysis of latent spatial representation to synchronize the representation of the original sequence, then input the random subset of the synchronous multivariate into an automatic encoder array that learns the loss of minimum quantile reconstruction, and finally infer and locate anomalies through majority voting. Liang et al. [20] proposed the multi-time-scale deep convolutional generative adversarial network (MTS-DCGAN) framework, which used cross-correlation calculation based on multi-time-scale sliding Windows to transform multivariate time-series into multi-channel feature matrix. By inputting the feature matrix into DCGAN, multi-layer CNN can capture the nonlinear interrelated features hidden in the original multivariate time-series without prior knowledge.

#### 2.1.2. Spatial Correlation-Based Method

Deng and Hooi [21] proposed an attention-based graph neural network method—GDN—which can learn the dependency graph between sensors, and identify and explain the deviation of these relationships, helping users infer the cause of anomalies. Chen, X. et al. [22] proposed GraphAD, a new multivariate time series anomaly detection model based on graph neural networks. They extracted patterns of key indicators from attribute, entity, and temporal perspectives via graph neural networks. Wang, Y. et al. [23] proposed a simple but effective graph self-supervised learning scheme called Deep Cluster Infomax for node representation learning, which captures intrinsic graph attributes in a more concentrated feature space by clustering the whole graph into multiple parts. Yang, J., and Yue, Z. [24] proposed a hierarchical spatial-temporal graph representation that constructs discriminative decision boundaries by learning hierarchical normality closed hyperspheres on the generated graph structural representation, without requiring a predefined topological prior. Razaque, A. et al. [25] proposed an anomaly detection paradigm called novel matrix profile to address the full-pair similarity search problem for time-series data in healthcare. A novel matrix profile can be used on large multivariate datasets and produces high-quality approximate solutions in a reasonable amount of time.

#### 2.1.3. The Method for Spatial-Temporal Correlation Fusion

Li, Y. et al. [26] proposed an inflated convolutional transformer-based GAN to improve the accuracy and generalization of the model. They utilized multiple generators and a single discriminator to mitigate the pattern collapse problem. Each generator consists of an inflated convolutional neural network and a transform module to obtain both fine-grained and coarse-grained information of the time series. Qin, S. et al. [27] proposed a novel method for time series anomaly detection based on transformer and signal decomposition. They provide a multi-view embedding method to capture temporal and correlation features of the signal. To make full use of temporal patterns, a frequency attention module is designed to extract periodic oscillation features. Kim, J. et al. [28] proposed an unsupervised prediction-based time series anomaly detection method using the transformer, which learns the dynamic patterns of sequential data through a self-attentive mechanism. The output representation of each transformer layer is accumulated in the encoder to obtain a representation with multiple levels and rich information. The decoder fuses this representation by means of a one-dimensional convolution operation. Therefore, the model can forecast while considering both the global trend and local variability of the input time series.

### 2.2. Short-Time Fourier Transform

In recent years, short-time Fourier transform has become a successful method for fault diagnosis or anomaly detection. In the existing research, most scholars have utilized the short-time Fourier transform to convert the signal into a spectrogram, then extract features from the spectrogram by using a convolutional neural network, and finally detect or classify abnormal signals. Gultekin et al. [29] proposed a data fusion method based on convolutional neural network, which used short-time Fourier transform to detect and identify operational faults in automatic transfer vehicles (ATVs). Li and Boulanger [30] combined the short-time Fourier transform spectra of the ECG signal with hand-made features to detect more complex cardiac abnormalities, including 16 distinct rhythm abnormalities and 13 heartbeat abnormalities. Zhou et al. [31] proposed a radio anomaly detection algorithm based on an improved GAN, which uses short-time Fourier transform to obtain the spectral graph image from the received signal, then reconstructs the spectral graph by combining the encoder network in the original GAN, and detects the anomaly according to the reconstruction error and discriminator loss. Chong et al. [32] studied the feasibility of detecting adverse substructure conditions by using bullet train load through finite element numerical simulation. All the synthesized signals obtained from the numerical simulation are analyzed using fast Fourier transform (FFT) in the frequency domain and short-time Fourier transform in the time-frequency domain. The three-dimensional Fourier scattering transform proposed by Kavalerov et al. [33] is a fusion of time-frequency representation and neural network architecture, taking advantage of short-time Fourier transform and the numerical computational efficiency of a deep learning network structure. Khan et al. [34] proposed a new network intrusion detection system (NIDS) framework based on deep convolutional neural networks, which utilized the network spectrum image generated by short-time Fourier transform to improve the accuracy of intrusion detection. Haleem et al. [35] used short-time Fourier transform to convert ECG beats into 2D images to automatically distinguish normal ECG from cardiac adverse events such as arrhythmia and congestive heart failure. Sanakkayala et al. [36] used short-time Fourier transform to convert bearing vibration signals into spectral graphs and then used the convolutional neural network VGG16 to extract features and classify health conditions.

As mentioned above, both deep learning techniques and short-time Fourier transform in multivariate time-series anomaly detection have shown superiority in some specific cases. In our paper, we combine the advantages of these two techniques to research multivariate time-series anomaly detection.

## 3. Methods

**Figure 1:**
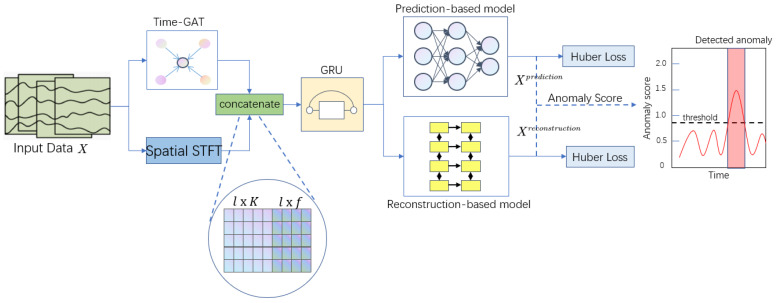
The overall architecture of our proposed DCFF-MTAD.

In this paper, we describe our multivariate time-series anomaly detection model DCFF-MTAD in detail. Figure 1 shows the general framework of DCFF-MTAD, which includes the following parts:

(1) Preprocessing module: multivariate time-series data collected from multiple sensors are normalized and used as input to the model; (2) dual-channel feature extraction module composed of time-based graph attention layer and short-term Fourier transform; (3) feature fusion module based on gated recurrent unit (GRU); (4) anomaly detection module composed of prediction model, reconstruction model, and anomaly score calculation method. Below is a detailed description of each of these modules.

### 3.1. Preprocessing Module

#### 3.1.1. Data Format

A univariate time-series is generated by a single sensor and is strictly arranged by timestamp. Multiple univariate time-series from the same entity form a multivariate time-series. In this paper, $t_{1},t_{2},\cdots ,t_{N}$ is used to represent the timestamp; there are *K* sensors in total, and the matrix *X* is used to represent the multivariate time-series as follows:

(1) $$X=\left[\begin{array}{cccc} x_{11} & x_{12} & \cdots & x_{1K}\\ x_{21} & x_{22} & \cdots & x_{2K}\\ \vdots & \vdots & \; & \vdots \\ x_{N1} & x_{N2} & \cdots & x_{NK} \end{array}\right]$$

where $x_{nk}\;\left(1\leq n\leq N,1\leq k\leq K\right)$ represents the value of the *k*th sensor at the timestamp $t_{n}$. In this paper, we use the sliding window of size l×K(1<l<N) to obtain data.

#### 3.1.2. Data Normalization

The input parameters of our model are multivariate data, which contain multiple variables with different dimensional units. In the training process, this can lead to low prediction accuracy of multivariate data, low reconstruction accuracy of multivariate data, and slow gradient descent of the optimal solution. To resolve these issues, the data normalization method is used to map the data values of multiple variables to the same scale. We use the maximum and minimum values of each sensor data for normalization:

(2) $${\tilde{x}}_{nk}=\frac{x_{nk}-x_{k}^{\min}}{x_{k}^{\max}-x_{k}^{\min}}$$

where $x_{k}^{\max}$ and $x_{k}^{\min}$ respectively represent the maximum and minimum values of the *k*th sensor data, and ${\tilde{x}}_{nk}$ represents the result of $x_{nk}$ after normalization processing.

### 3.2. Spatial Short-Time Fourier Transform

The curve forms of multivariate data are complex and varied, and most of the curves are non-stationary signals. Since time-domain methods cannot obtain frequency information, and frequency-domain methods cannot obtain instantaneous features, time-frequency transform methods are often used in non-stationary signal analysis to diagnose industrial machinery faults [37]. STFT is a conventional and classic linear time-frequency analysis method, which overcomes the shortcomings of the traditional Fourier transform, which cannot reveal the local characteristics of the signal. Unlike STFT, the Wigner distribution is a nonlinear distribution. Since the Wigner distribution does not use a window function similar to the STFT definition, it has no loss of resolution when analyzing signals. Wigner distribution is a traditional quadratic time-frequency distribution, and suffers from the cross-terms presence, which will generate redundant interference information when processing multi-component signals. The cross-terms-free Wigner distribution (frequently named the S-method) unifies the desirable properties of STFT (cross-terms-free nature) and Wigner distribution (optimal auto-terms presentation, high concentration, resolution, and selectivity). Besides, the S-method provides both the noise influence reduction in comparison to the conventional time-frequency tools (the spectrogram and the Wigner distribution) [38] and the best performances in estimation of the instantaneous frequency [39]. Compared with STFT, the operation of S-method is more complex, and it needs more computation when dealing with multivariate data. Although the S-method has better time-frequency analysis performance, its more complex computation is not conducive to practical engineering applications. Considering the advantages and disadvantages of various methods, STFT is used to extract features from a large number of multivariate time series data.

**Figure 2:**
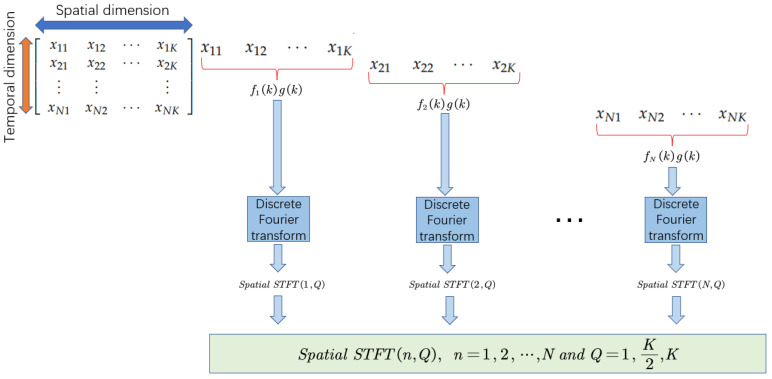
The calculation process of spatial STFT.

Unlike the previous approach of applying STFT in the time dimension, we apply STFT in the spatial dimension of multivariate data. Figure 2 illustrates how we perform the short time Fourier transform in the spatial dimension.

The spatial short-time Fourier transform is defined as follows:

(3) $$\;\mathrm{Spatial}\;\mathrm{STFT}\left(n,Q\right)=\mathrm{DFT}\left\{f_{n}\left(k\right)g\left(k\right)\right\},n=1,2,\cdots N$$

where DFT denotes discrete Fourier transform. $f_{n}\left(k\right)$ denotes a function formed by multiple variables in the spatial dimension and g(k) denotes a window function. *Q* denotes the location of the computed Fourier transform.

The spatial short-time Fourier transform is carried out on the multivariate time-series data whose tensor dimension is (l,K). Set the window size (nperseg) of STFT to the dimension of the dataset and the number of window function overlap to the default value of 50%. After the transformation, the three-dimensional tensor with dimension (l,f,t) is obtained. *f* denotes the dimension of the extracted spatial features and *t* denotes the dimension of the centroid of the window function.

### 3.3. Time-Based Graph Attention Layer (Time-GAT)

The graph attention network (GAT) uses attention weights to replace connections between nodes that are either 0 or 1 and is able to automatically learn and optimize connections between nodes by using the attention mechanism. The relationship between nodes can be optimized into continuous values so that the expression of information is more abundant, and the correlation between nodes can be better integrated into the model. The graph attention network does not use the Laplace matrix for complex calculation, and the calculation of its attention value is carried out in parallel between nodes, so the calculation process of the graph attention network is very efficient. In this paper, we use the same method as Zhao et al. [40] to construct the graph attention layer based on time.

**Figure 3:**
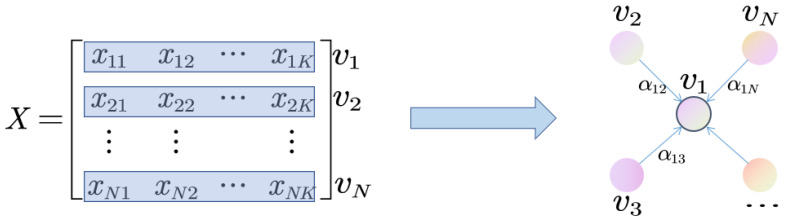
Schematic diagram of constructing a time-based graph attention layer.

As can be seen in Figure 3, all the data in the sliding window is considered to be a complete graph. Specifically, the feature vector at a timestamp is represented by a node, and the relationship between two timestamps is represented by an edge. In this way, the time dependence in the time-series data can be captured.

Generally, given a graph with *N* nodes, i.e., $\left\{v_{1},v_{2},\cdots ,v_{N}\right\}$, where $v_{i}$ represents the feature vector of each node, the calculated output of each node is as follows:

(4) $$h_{i}=\sigma \left(\sum_{j=1}^{L}\alpha_{ij}v_{j}\right)$$

where σ represents the sigmoid activation function. $\alpha_{ij}$ represents the attention score that measures the contribution of node *j* to node *i*, and node *j* is one of the adjacent nodes of node *i*. *L* represents the total number of adjacent nodes of node *i*.

The calculation formula of the attention score $\alpha_{ij}$ is as follows:

(5) $$e_{ij}=LeakyReLU\left(\omega^{T}\cdot \left(v_{i}⊕v_{j}\right)\right)$$

(6) $$\alpha_{ij}=\frac{\exp\left(e_{ij}\right)}{\sum_{l=1}^{L}\exp\left(e_{il}\right)}$$

where ⊕ represents the concatenation of two node representations, $\omega \in R^{2m}$ is a column vector of learnable parameters, and *m* represents the dimension of the feature vector of each node.

### 3.4. Feature Fusion Module Based on GRU

The dimension of the output data of the time-based graph attention layer is (l,K), and the dimension of the output data after spatial short-time Fourier transform is (l,f,t). Since the data after the short-time Fourier transform is a complex number, we conduct modulo operation on it. To match the dimensions of the output data of the two channels, we conduct average operation on the third dimension *t* of the three-dimensional tensor (l,f,t). Finally, we get the two-dimensional tensor (l,f).

The GRU is a variant of the traditional RNN, which can effectively capture the correlation between time-series data and alleviate the phenomenon of gradient disappearance or gradient explosion in traditional neural networks. Compared with LSTM network, GRU has a simpler structure and fewer parameters and requires less time in the training phase. Therefore, GRU is chosen as the model of the feature fusion module.

**Figure 4:**
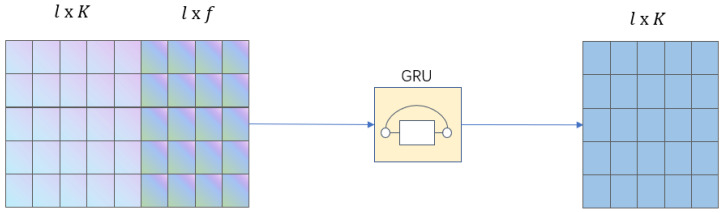
Feature fusion map.

The time and spatial features of the multivariate time-series are respectively extracted through the time-based graph attention layer and the spatial short-time Fourier transform. In this paper, the output data of the two channels are concatenated in the way shown in Figure 4 to obtain a tensor with dimension (l,K+f), which is then sent to the GRU and mapped into a tensor with dimension (l,K) through the GRU.

### 3.5. Loss Function

In multivariate time-series anomaly detection, loss function plays a very important role. The loss function is a function used to measure the gap between the predicted data and the actual data. For the same neural network, the selection of loss function will affect the quality of model training to a certain extent.

Huber Loss is a piece-wise loss function for regression problems, which combines the advantages of both mean absolute error (MAE) and mean square error (MSE). A model using MSE as a loss function is likely to forcibly fit outliers to reduce the value of the loss function, thereby affecting the output of the model. Compared with MSE, Huber Loss has better robustness to outliers. Huber Loss is selected for its reliability and validity, which is defined as follows:

(7) $$L_{\delta }\left(y,\hat{y}\right)=\left\{\begin{array}{c} \frac{1}{2}{\left(y-\hat{y}\right)}^{2},\;\left|y-\hat{y}\right|⩽\delta \\ \delta |y-\hat{y}|-\frac{1}{2}\delta^{2},\;\left|y-\hat{y}\right|>\delta \end{array}\right.$$

where $\hat{y}$ represents the predicted value of *y*, and δ is a boundary. When the absolute value of the difference between the actual value and the predicted value is less than or equal to δ, the square error is used; When the absolute value of the difference between the actual value and the predicted value is greater than δ, Loss is reduced and a linear function is used. This approach can reduce the weight of outliers in the calculation of Loss and prevent overfitting of the model.

A prediction-based model is used to predict the value of the next timestamp. We use the same approach as Zhao et al. [40], stacking three fully-connected layers after GRU as the prediction-based model. A reconstruction-based model is used to capture the data distribution across the time-series. We use a GRU-based decoder as the reconstruction-based model.

During training, the parameters of the prediction-based and reconstruction-based models are updated simultaneously. Both models use Huber Loss as the loss function, and the loss function of the entire model is defined as the sum of the loss functions of the two models, as shown in the following formula.

(8) $$Loss=Loss_{\mathrm{prediction}\;}+Loss_{\mathrm{reconstruction}\;}$$

where $Loss_{\mathrm{prediction}\;}$ represents the loss function of the prediction-based model and $Loss_{\mathrm{reconstruction}\;}$ represents the loss function of the reconstruction-based model.

### 3.6. Anomaly Determination

There are two results for each timestamp: one is the predicted value based on the prediction model and the other is the reconstructed value based on the reconstruction model. We calculate the anomaly score for each variable and take the average of the anomaly scores for all variables as the final anomaly score. The final anomaly score is calculated as follows:

(9) $$\;\mathrm{Anomaly}\;\mathrm{Score}\;=\frac{1}{K}\sum_{k=1}^{K}\left(\left|x_{k}^{\mathrm{prediction}\;}-x_{k}\right|+\gamma \left|x_{k}^{\mathrm{reconstruction}\;}-x_{k}\right|\right)$$

where $\left|x_{k}^{\mathrm{prediction}\;}-x_{k}\right|$ represents the deviation degree between the predicted value and the actual value of variable *k* and $\left|x_{k}^{\mathrm{reconstruction}\;}-x_{k}\right|$ represents the deviation degree between the reconstructed value and the actual value of variable *k*. *K* is the total number of variables. γ is a hyperparameter that balances the error based on the prediction model with the error based on the reconstruction model.

The threshold is set to give the best boundary between normal and abnormal data. Hundman et al. [15] proposed a non-parametric dynamic threshold method, which is an unsupervised method and does not depend on labeled data and statistical assumptions about errors. The non-parametric dynamic threshold method is selected for its low computational cost and high performance. We calculate the threshold for each variable and take the average of the thresholds for all variables as the final threshold. If the anomaly score on a timestamp is greater than the final threshold, the timestamp is marked as an anomaly.

## 4. Experiment

We have trained and evaluated DCFF-MTAD on three publicly available datasets, compared it with existing state-of-the-art models for detecting anomalies in multivariate time-series, and analyzed the experimental results. The datasets, evaluation metrics, implementation details, comparative experiments, ablation study, and sensitivity analysis are presented in this section.

### 4.1. Datasets

We selected three publicly available datasets, including soil moisture active passive (SMAP) [41], server machine dataset (SMD) [18], and mars science laboratory (MSL) [41], which are widely used anomaly detection datasets.

SMAP is a publicly available dataset of the amount of water in the earth’s topsoil collected by NASA. The observation mission uses both active and passive sensors. The active sensor is the L-band radar, and the passive sensor is the L-band microwave radiometer.

SMD is a 5-week public dataset collected from 28 computers of a large Internet company.

MSL is a publicly available dataset from NASA that contains telemetry data from spacecraft monitoring systems for unexpected event anomaly reports.

Table 1 shows the detailed statistics of the three datasets, including dataset name, number of entities, dimensionality, size of training set and test set, and the ratio of anomalies in the test set.

### 4.2. Evaluation Metrics

We evaluate the anomaly detection performance of all models using precision, recall, and F1-score on the test dataset. Precision indicates the percentage of correctly detected anomalies among all detected anomalies, recall indicates the percentage of correctly detected anomalies among all anomalies, and the F1 value is the harmonic mean of precision and recall.

(10) $$\mathrm{Precision}=\frac{\mathrm{TP}}{\mathrm{TP}\;+\;\mathrm{FP}}$$

(11) $$\mathrm{Recall}=\frac{\mathrm{TP}}{\mathrm{TP}\;+\;\mathrm{FN}}$$

(12) $$F1=\frac{2\times \mathrm{Precision}\times \mathrm{Recall}}{\mathrm{Precision}\;+\;\mathrm{Recall}}$$

where TP represents the number of samples correctly detected as abnormal in abnormal samples; TN represents the number of samples correctly detected as normal in normal samples; FP represents the number of samples correctly detected as abnormal in normal samples; FN represents the number of samples correctly detected as normal in abnormal samples.

### 4.3. Comparative Experiments

#### 4.3.1. Implementation Details

We implemented our model in Pytorch 1.10.0 and CUDA 10.2. The model was fully trained on a server equipped with Intel(R) Xeon(R) Silver 4110 CPU @2.10GHz and an NVIDIA Tesla P100 GPU (16G memory). To be fair, we set the learning rate for all models to 0.001, using the Adam optimizer for 10 training sessions and the non-parametric dynamic threshold method. In our model, we set γ to 1, the batch size to 256, and the hidden layer dimensions of the GRU-based feature fusion module, the prediction-based model and the reconstruction-based model are all 150. In the feature fusion module based on GRU, the prediction-based model, and the reconstruction-based model, the dropout mechanism [42] is used to prevent the overfitting problem of the complex model. The key idea is to drop some neurons (set output to zero) randomly during the training process with some probability, which helps to prevent complex co-adaptations on training data. The dropout ratio adopted in this paper is 0.3.

#### 4.3.2. Experimental Results

We compared DCFF-MTAD with existing state-of-the-art models for multivariate time-series anomaly detection, including LSTM [15], OmniAnomaly [18], USAD [17], MAD-GAN [16], DAGMM [14], and GDN [21]. Table 2 shows the F1, precision (P), and recall (R) of all models on the three public datasets. In terms of the F1 value, compared with existing state-of-the-art models, our network structure performs well in multivariate time-series anomaly detection, especially on the SMAP and SMD datasets. On the MSL dataset, OmniAnomaly shows the best anomaly detection performance, but our model is also second only to OmniAnomaly and outperforms the remaining models.

As can be seen from Table 2, LSTM’s performance is suboptimal because it only captures time information, as indicated by its F1 values of 0.5800 and 0.8322 on the SMAP and MSL datasets, respectively. MAD-GAN’s performance is unstable, with F1 values of 0.5725 and 0.8367 on the SMAP and MSL datasets, respectively, due to the difficulty of training GAN-based network, which may suffer from issues such as mode collapse and non-convergence. USAD performs well on the SMAP, SMD, and MSL datasets, with F1 values of 0.8360, 0.8704, and 0.8959, respectively. USAD’s encoder-decoder architecture combines the advantages of autoencoders and adversarial training, and is able to learn how to amplify the reconstruction error of inputs containing anomalies. USAD is more stable and robust than methods based on GAN architectures. The F1 value of DAGMM on MSL dataset is 0.9351, which is lower than that of OmniAnomaly and Our Model. DAGMM saves the key information of input samples in low-dimensional space, including the features discovered through dimensionality reduction and reconstruction errors, so it shows good performance on MSL dataset with higher dimensions. F1 values of GDN on SMAP, SMD, and MSL datasets are 0.5773, 0.7486, and 0.9051, respectively. The GDN captures the unique characteristics of each sensor, and its graph structure can learn the relationships between high-dimensional sensors to detect deviations in those relationships. The higher the dimension of the dataset, the better the GDN can play its advantages, and the higher its F1 value. The F1 values of OmniAnomaly on the SMD and MSL datasets are 0.8709 and 0.9509, respectively. OmniAnomaly uses stochastic recurrent neural networks to model the explicit time dependence between random variables, and planar normalization streams to better capture the complex distribution of input data. Therefore, OmniAnomaly shows good performance on the more complex high-dimensional MSL dataset. LSTM focuses on the temporal features of multivariate data, and GDN focuses on the spatial features of multivariate data. Our model extracts the spatial and temporal features of multivariate data through spatial STFT and time-based graph attention layer, and these two features are further fused. Therefore, our model contains richer feature information, which is an important reason why our model is superior to other models. Our model improves F1 values by 7.12% and 4.95% on SMAP and SMD datasets, respectively. In addition, Huber Loss is used to improve the robustness of the model, which makes the performance of our model on the three datasets very stable. Although our model performs well on F1, the complexity of our model is high. As shown in Table 3, parameters, FLOPs, and runtime of our model are larger than other models.

### 4.4. Ablation Study

In this section, we conducted a series of experiments to verify the effectiveness of the different components in the proposed DCFF-MTAD.

#### 4.4.1. Effectiveness of Dual-Channel Feature Extraction Module

We propose a dual-channel module to extract features from multivariate time-series data, which makes the feature information richer and improves anomaly detection accuracy. To further demonstrate the effectiveness of our proposed dual-channel feature extraction module, we conducted a series of experiments, including single-channel, dual-channel, and triple-channel, and the experimental results are shown in Table 4. The feature-based graph attention layer (Feat-GAT) in the ablation experiment was constructed in the same way as in the study of Zhao et al. [40].

On the SMAP dataset, our model shows better detection performance than the network composed of spatial STFT and Feat-GAT, with 33.06% improvement of the F1 value. Although the F1 value of our model is only 0.19% higher than that of the triple-channel network, our model has fewer parameters, less computation, and shorter training time.

On the SMD dataset, our model performs better than the network composed of spatial STFT and Feat-GAT, and the F1 value is improved by 3.35%. Our model performs better than the triple-channel network, and the F1 value increases by 4.53%.

On the MSL dataset, our model performs weaker than the network composed of spatial STFT and Feat-GAT, because the MSL dataset has a higher dimension than SMAP and SMD. A major advantage of Feat-GAT is that it can capture the relationship between higher-dimensional data. Therefore, Feat-GAT has given full play to its advantage on the MSL dataset. Compared with the triple-channel network, our model still performs well, and the F1 value has increased by 1.17%.

The experimental results of the above three public datasets show that our dual-channel feature extraction module can extract richer data information, both in the time dimension and in the spatial dimension. Therefore, our dual-channel feature extraction module achieves better detection results than single-channel networks. In addition, our dual-channel feature extraction module has fewer parameters than the triple-channel network, which shortens the training time of the model.

#### 4.4.2. Effectiveness of Huber Loss

In this section, we investigate the effect of Huber Loss on model performance. To verify the effectiveness of Huber Loss, we choose ordinary MSE Loss as the baseline. Above the baseline, MSE Loss is replaced by Huber Loss, and other modules in the model remain unchanged. We conduct this ablation experiment on three public datasets, and Table 5 shows the results of the ablation experiments.

When Huber Loss is used as the loss function of the model, the detection index is significantly improved. On the SMAP dataset, F1 value, precision, and recall are increased by 5.59%, 3.27%, and 7.57%, respectively. On the SMD dataset, the F1 value and precision are increased by 2.55% and 4.69%, respectively. On the MSL dataset, the F1 value and precision are improved by 0.85% and 1.69%, respectively. The experimental results show that Huber Loss as a loss function can improve the detection performance of the model.

#### 4.4.3. Effectiveness of Threshold Calculation Method

A study [43] showed that determining an appropriate threshold is as important as the algorithm itself. The experiments in the part will further prove the point. In multivariate time-series anomaly detection, there are two common threshold calculation methods: non-parametric dynamic threshold (NDT) and peak over threshold (POT) [44]. We used the two threshold calculation methods to conduct experiments on three public datasets, and Table 6 shows the experimental results.

When NDT is used as threshold calculation method, the detection metrics are significantly improved. On the SMD dataset, the F1 value and precision increased by 28.07% and 51.67%, respectively. On the MSL dataset, the F1 value and recall increased by 25.43% and 57.78%, respectively. The experimental results show that NDT can significantly improve the detection performance of the model.

### 4.5. Sensitivity Analysis

The model proposed in this paper involves many parameters, such as sliding window size, learning rate, γ, batch size, and the hidden layer dimensions of the GRU-based feature fusion module, the prediction-based model, and the reconstruction-based model. Sliding window size and γ are more important than other parameters. Therefore, we focus on analyzing the sensitivity of the sliding window size and γ.

#### 4.5.1. Sensitivity to the Window Size

**Figure 5:**
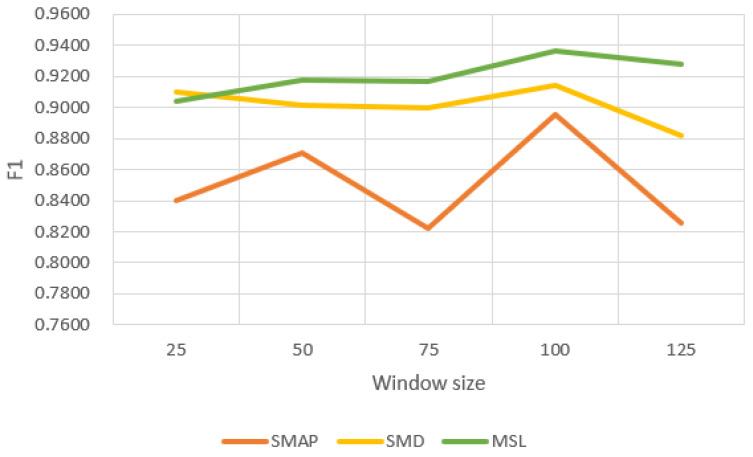
F1 score with window size.

**Figure 6:**
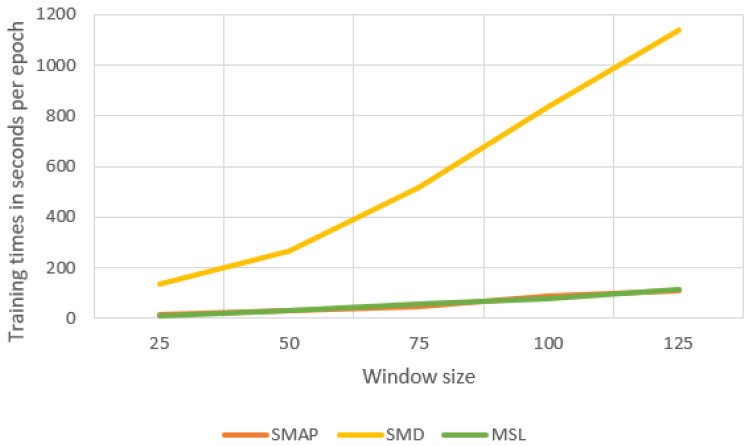
Training times with window size.

The sliding window size is an important parameter that affects model performance and training time. We conducted several sets of experiments with different window sizes on three public datasets, and the experimental results are shown in Figure 5 and Figure 6. Compared with SMAP, F1 values fluctuate less on SMD and MSL. When we use smaller windows, our model takes less time to train. If the window is too small, our model cannot capture local contextual information well. If the window is too large, the training time of the model will increase. The window size of 100 used in the experiments balances the F1 score and training time.

#### 4.5.2. Sensitivity to γ

**Figure 7:**
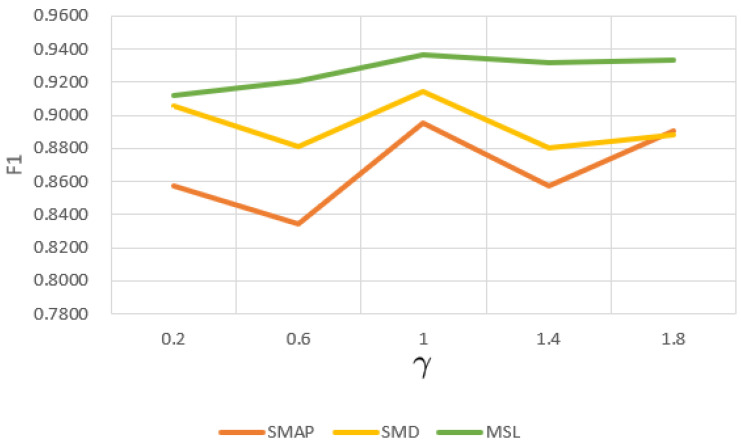
F1 score with γ.

γ is an important parameter used to balance the error of the prediction model and the error of the reconstruction model. We conducted several experiments with different γ values on three public datasets, and the experimental results are shown in Figure 7. Compared with MSL, the F1 values fluctuate more on SMAP and SMD. The γ of 1 we use in the experiments balances prediction-based and reconstruction-based models well.

## 5. Case Study

In this section, we verify the effectiveness and practicality of DCFF-MTAD on our dataset, which focuses on the shield machine—a critical piece of engineering equipment used for tunnel excavation in projects such as subways and highway tunnels. Due to the complexity and uncertainty of the underground environment, engineering safety and quality problems occur from time to time. For our study, we consider a tunnel project section with a total length of 943.5 m, where the shield tunneling method is employed for construction. Specifically, we analyze an abnormal event report which indicates that the shield machine passed through a karst cave between 13:23 and 21:32 on 2 November 2020. The geological survey report shows that the cave fillings consist of soft plastic silty clay, gravel sand, gravel, and other materials, which pose significant challenges for anomaly detection due to their inhomogeneity and complexity.

We used 31,333 pieces of normal data from 13:35 on 23 October 2020 to 14:08 on 29 October 2020 as training data, and 6475 pieces of data from 06:01 on 2 November 2020 to 21:56 on 2 November 2020 as testing data. We selected the total thrust, advance speed, cutter head torque, and screw speed recorded in the abnormal event report as the input parameters of our model. Firstly, the input parameters were normalized, and then the processed data were input into the spatial STFT module and the time-based graph attention layer in parallel to extract the spatial and temporal features of the input parameters, respectively. The output representations of the spatial STFT module and the time-based graph attention layer were concatenated and fed into the GRU to fuse spatial and temporal features. The output of the GRU was fed into the prediction-based and reconstruction-based models in parallel to obtain the predicted and reconstructed values of the input parameters.

**Figure 8:**
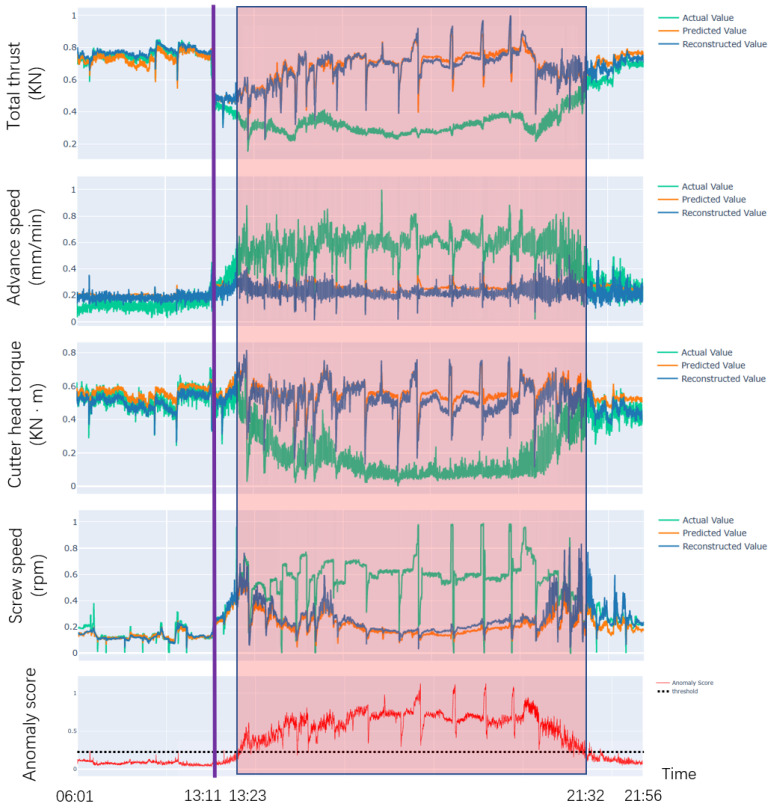
Anomaly detection result graph.

The experimental results on the testing data are shown in Figure 8. The green curve represents the actual value, the orange curve represents the predicted value obtained by the prediction-based model, and the dark blue curve represents the reconstructed value obtained by the reconstruction-based model, all of which are normalized values. Our dual-channel feature extraction module fully extracts the time and spatial features of the construction parameters, and the prediction and reconstruction models use these two features to predict and reconstruct the data. We used the anomaly score to measure the difference between the value obtained by the model and the actual value, and an appropriate threshold was calculated using the non-parametric dynamic threshold method. When the anomaly score consistently exceeds the threshold, we consider it an abnormal phenomenon, which is indicated by the light red box.

As shown in Figure 8, the time of the purple line is 13:11 on 2 November 2020. From 06:01 to 13:11, total thrust, advance speed, cutter head torque, and screw speed of the shield machine fluctuated around 0.75, 0.1, 0.5, and 0.1, respectively, and the anomaly score was always below the threshold. From 13:11 to 13:23, the shield machine gradually approached the cave area, total thrust showed a downward trend, advance speed and screw speed showed an upward trend, cutter head torque showed obvious fluctuations, and the anomaly score gradually increased but did not exceed the threshold. From 13:23 to 21:32, when the shield machine tunneled forward in the cave area, the total thrust and cutter head torque decreased, the advance speed and screw speed increased, and the anomaly score exceeds the threshold—this period is marked as abnormal. After 21:32, the shield machine left the cave area and the four construction parameters gradually returned to the normal range, with the anomaly score gradually falling below the threshold.

Our model extracts both spatial and temporal features of the construction parameters through spatial STFT and time-based graph attention layer. The two features are fused. This is because our model extracts the rich information hidden in the data, so the model successfully can learn the normal behavior pattern of construction parameters. When the construction parameter is abnormal, our model can detect the anomaly quickly. From this, it can be seen that DCFF-MTAD is able to monitor whether the shield machine is operating normally or not without much prior knowledge of the detection of abnormal behavior. This would be of great significance for ensuring the safe operation of the shield machine and improving its reliability by combining the proposed method with the automatic intelligent control technology of the shield machine.

## 6. Conclusions and Future Work

In this paper, we propose DCFF-MTAD, a stable and practical multivariate time series anomaly detection model, which employs dual-channel feature fusion. The performance of the model is mainly guaranteed by (1) the temporal features of multivariate data extracted by time-based graph attention layer; (2) the spatial features of multivariate data extracted by spatial STFT; (3) the feature-level fusion of temporal features and spatial features enriches the feature representation of data; and (4) the introduction of Huber Loss makes the anomaly detection performance of the model more robust. Extensive experiments are conducted to tune and validate the hyperparameters of the model to achieve the best performance. Experimental results show that compared with the state-of-the-art approaches, our method has the best anomaly detection performance on the SMAP and SMD public datasets, with 7.12% and 4.95% improvement in F1 values, respectively. The F1 score of our method is second only to OmniAnomaly on the MSL public dataset. In a practical application scenario, our method can continuously monitor important parameters during shield tunneling. Anomalies in construction parameters can be well detected when the parameters deviate from normal behavior.

In future research, we will improve our method so that it can be widely used in multivariate time series anomaly detection. The following suggestions can further improve the performance of the method and increase the availability of the method in anomaly detection: (1) The fully connected neural network used in the prediction-based model and the GRU used in the reconstruction-based model are more commonly used methods. If lower model complexity is required, the computational efficiency and performance of the entire model could be improved by replacing them with faster and better modules. (2) The choice of threshold is very important for the anomaly detection performance of the model. If the selected threshold is too high, only a few anomalies can be detected; if the selected threshold is too low, normal samples will be misjudged as abnormal. To further improve the anomaly detection performance and universality of the model, two existing threshold calculation methods (peak over threshold and non-parametric dynamic threshold) can be optimized or a new one can be developed. (3) In the case study, our model learned features from the normal behavior of construction parameters. On small-scale datasets, unsupervised learning may be extended to supervised learning, which means that both normal behavior and abnormal behavior can be used for learning data features.

## Figures and Tables

**Table 1:** Dataset statistics.

Dataset Name	Number of Entities	Number of Dimensions	Training Set Size	Testing Set Size	Anomaly Ratio (%)
SMAP	55	25	135,183	427,617	13.13
SMD	28	38	708,405	708,420	4.16
MSL	27	55	58,317	73,729	10.72

**Table 2:** Anomaly detection performance metrics on three datasets. The best results are highlighted in bold.

Models	SMAP			SMD			MSL		
	F1	P	R	F1	P	R	F1	P	R
LSTM	0.5800	0.9809	0.4118	0 *	0 *	0 *	0.8322	0.7126	0.9999
OmniAnomaly	0.5789	0.9747	0.4118	0.8709	0.7713	0.9999	**0.9509**	0.9064	0.9999
USAD	0.8360	0.9734	0.7326	0.8704	0.7713	0.9989	0.8959	0.8115	0.9999
MAD-GAN	0.5725	0.9390	0.4118	0 *	0 *	0 *	0.8367	0.7193	0.9999
DAGMM	0.5752	0.9536	0.4118	0.7892	0.6518	0.9999	0.9351	0.8782	0.9999
GDN	0.5773	0.9655	0.4118	0.7486	0.5991	0.9974	0.9051	0.8267	0.9999
Our Model	**0.8955**	0.9767	0.8268	**0.9140**	0.8416	0.9999	0.9366	0.9257	0.9478

* The place of 0 in this table is because the threshold selected by the non-parametric dynamic threshold method is too high, resulting in TP and FP being 0. Therefore, the three calculation indicators are all 0.

**Table 3:** Model complexity.

Model	Parameters	FLOPs	Runtime
LSTM	3962	20,120	1.8 ms
OmniAnomaly	18,490	37,760	3.8 ms
USAD	10,609	33,760	3.7 ms
MAD_GAN	9903	25,920	3.1 ms
DAGMM	10,516	21,234	3.4 ms
GDN	4160	61,956	13.9 ms
Our Model	325,934	3,207,080	51 ms

**Table 4:** The effects of dual-channel feature extraction module on three datasets. The best results are highlighted in bold. 🗸 indicates that the module is used for experiments.

STFT	Time-GAT	Feat-GAT	SMAP			SMD			MSL		
			F1	P	R	F1	P	R	F1	P	R
🗸			0.8086	0.9242	0.7187	0.9019	0.8213	0.9999	0.9196	0.9048	0.9348
	🗸		0.8060	0.9759	0.6864	0.8335	0.7146	0.9999	0.8952	0.9037	0.8869
🗸	🗸		**0.8955**	0.9767	0.8268	**0.9140**	0.8416	0.9999	0.9366	0.9257	0.9478
🗸		🗸	0.6730	0.9866	0.5106	0.8844	0.7928	0.9999	**0.9542**	0.9662	0.9426
🗸	🗸	🗸	0.8938	0.9588	0.8372	0.8744	0.7768	0.9999	0.9258	0.9607	0.8934

**Table 5:** The effects of Huber Loss. The best results are highlighted in bold.

Loss	SMAP			SMD			MSL		
	F1	P	R	F1	P	R	F1	P	R
MSE	0.8481	0.9458	0.7686	0.8913	0.8039	0.9999	0.9287	0.9103	0.9478
Huber	**0.8955**	0.9767	0.8268	**0.9140**	0.8416	0.9999	**0.9366**	0.9257	0.9478

**Table 6:** The effects of the threshold calculation method. The best results are highlighted in bold.

Method	SMAP				SMD				MSL			
	F1	P	R	Threshold	F1	P	R	Threshold	F1	P	R	Threshold
POT	0 *	0 *	0 *	1.0967	0.7137	0.5549	0.9999	0.1424	0.7467	0.9865	0.6007	0.9086
NDT	**0.8955**	0.9767	0.8268	0.6390	**0.9140**	0.8416	0.9999	0.2111	**0.9366**	0.9257	0.9478	0.5845

* On the SMAP dataset, the threshold obtained by POT is too high, resulting in TP and FP being 0. Therefore, the three calculation indicators are all 0.

## Data Availability

This research employed publicly available datasets for its experimental studies. The data in the case study are not publicly available due to the confidentiality requirement of the project.

## Abbreviations

The following abbreviations are used in this manuscript:

STFT	Short-time Fourier Transform
RNN	Recurrent Neural Network
TCN	Temporal Convolutional Network
GNN	Graph Neural Network
DAGMM	Deep Autoencoder Gaussian Mixture Model
LSTM	Long Short-Term Memory
NDT	Non-parametric Dynamic Threshold
GAN	Generative Adversarial Network
GDN	Graph Deviation Network
USAD	UnSupervised Anomaly Detection
MTS-DCGAN	Multi-Time-Scale Deep Convolutional Generative Adversarial Network
CNN	Convolutional Neural Network
ATV	Automatic Transfer Vehicle
ECG	electrocardiogram
FFT	Fast Fourier Transform
NIDS	Network Intrusion Detection System
VGG	Visual Geometry Group
GRU	Gated Recurrent Unit
GAT	Graph Attention Network
MAE	Mean Absolute Error
MSE	Mean Square Error
SMAP	Soil Moisture Active Passive
SMD	Server Machine Dataset
MSL	Mars Science Laboratory
NASA	National Aeronautics and Space Administration
MAD	Multivariate Anomaly Detection
FLOPs	floating point operations
POT	Peak Over Threshold

## References

[1] Ismail Fawaz H., Forestier G., Weber J., Idoumghar L., Muller P.A. (2019). Deep learning for time series classification: A review. Data Min. Knowl. Discov..

[2] Chauhan S., Vig L. Anomaly detection in ECG time signals via deep long short-term memory networks. Proceedings of the IEEE International Conference on Data Science and Advanced Analytics (DSAA).

[3] Goh J., Adepu S., Tan M., Lee Z.S. Anomaly detection in cyber physical systems using recurrent neural networks. Proceedings of the 2017 IEEE 18th International Symposium on High Assurance Systems Engineering (HASE).

[4] Ding N., Ma H., Gao H., Ma Y., Tan G. (2019). Real-time anomaly detection based on long short-Term memory and Gaussian Mixture Model. Comput. Electr. Eng..

[5] Wu W., He L., Lin W., Su Y., Cui Y., Maple C., Jarvis S. (2020). Developing an unsupervised real-time anomaly detection scheme for time series with multi-seasonality. IEEE Trans. Knowl. Data Eng..

[6] Shen L., Li Z., Kwok J. (2020). Timeseries anomaly detection using temporal hierarchical one-class network. Adv. Neural Inf. Process. Syst..

[7] He Y., Zhao J. (2019). Temporal convolutional networks for anomaly detection in time series. J. Phys. Conf. Ser..

[8] Chen Z., Chen D., Zhang X., Yuan Z., Cheng X. (2021). Learning graph structures with transformer for multivariate time-series anomaly detection in IoT. IEEE Internet Things J..

[9] Dai E., Chen J. (2022). Graph-augmented normalizing flows for anomaly detection of multiple time series. arXiv.

[10] Han S., Woo S.S. Learning Sparse Latent Graph Representations for Anomaly Detection in Multivariate Time Series. Proceedings of the 28th ACM SIGKDD Conference on Knowledge Discovery and Data Mining.

[11] Xu J., Wu H., Wang J., Long M. (2021). Anomaly transformer: Time series anomaly detection with association discrepancy. arXiv.

[12] Tuli S., Casale G., Jennings N.R. (2022). Tranad: Deep transformer networks for anomaly detection in multivariate time series data. arXiv.

[13] Wang X., Pi D., Zhang X., Liu H., Guo C. (2022). Variational transformer-based anomaly detection approach for multivariate time series. Measurement.

[14] Zong B., Song Q., Min M.R., Cheng W., Lumezanu C., Cho D., Chen H. Deep autoencoding gaussian mixture model for unsupervised anomaly detection. Proceedings of the International Conference on Learning Representations.

[15] Hundman K., Constantinou V., Laporte C., Colwell I., Soderstrom T. Detecting spacecraft anomalies using lstms and nonparametric dynamic thresholding. Proceedings of the 24th ACM SIGKDD International Conference on Knowledge Discovery & Data Mining.

[16] Li D., Chen D., Jin B., Shi L., Goh J., Ng S.K. (2019). MAD-GAN: Multivariate anomaly detection for time series data with generative adversarial networks. Proceedings of the Artificial Neural Networks and Machine Learning–ICANN 2019: Text and Time Series: 28th International Conference on Artificial Neural Networks.

[17] Audibert J., Michiardi P., Guyard F., Marti S., Zuluaga M.A. Usad: Unsupervised anomaly detection on multivariate time series. Proceedings of the 26th ACM SIGKDD International Conference on Knowledge Discovery & Data Mining.

[18] Su Y., Zhao Y., Niu C., Liu R., Sun W., Pei D. Robust anomaly detection for multivariate time series through stochastic recurrent neural network. Proceedings of the 25th ACM SIGKDD International Conference on Knowledge Discovery & Data Mining.

[19] Abdulaal A., Liu Z., Lancewicki T. Practical approach to asynchronous multivariate time series anomaly detection and localization. Proceedings of the 27th ACM SIGKDD Conference on Knowledge Discovery & Data Mining.

[20] Liang H., Song L., Wang J., Guo L., Li X., Liang J. (2021). Robust unsupervised anomaly detection via multi-time scale DCGANs with forgetting mechanism for industrial multivariate time series. Neurocomputing.

[21] Deng A., Hooi B. Graph neural network-based anomaly detection in multivariate time series. Proceedings of the AAAI Conference on Artificial Intelligence.

[22] Chen X., Qiu Q., Li C., Xie K. GraphAD: A Graph Neural Network for Entity-Wise Multivariate Time-Series Anomaly Detection. Proceedings of the 45th International ACM SIGIR Conference on Research and Development in Information Retrieval.

[23] Wang Y., Zhang J., Guo S., Yin H., Li C., Chen H. Decoupling representation learning and classification for gnn-based anomaly detection. Proceedings of the 44th International ACM SIGIR Conference on Research and Development in Information Retrieval.

[24] Yang J., Yue Z. (2022). Learning Hierarchical Spatial-Temporal Graph Representations for Robust Multivariate Industrial Anomaly Detection. IEEE Trans. Ind. Inform..

[25] Razaque A., Abenova M., Alotaibi M., Alotaibi B., Alshammari H., Hariri S., Alotaibi A. (2022). Anomaly detection paradigm for multivariate time series data mining for healthcare. Appl. Sci..

[26] Li Y., Peng X., Zhang J., Li Z., Wen M. (2021). DCT-GAN: Dilated convolutional transformer-based gan for time series anomaly detection. IEEE Trans. Knowl. Data Eng..

[27] Qin S., Zhu J., Wang D., Ou L., Gui H., Tao G. Decomposed Transformer with Frequency Attention for Multivariate Time Series Anomaly Detection. Proceedings of the 2022 IEEE International Conference on Big Data (Big Data).

[28] Kim J., Kang H., Kang P. (2023). Time-series anomaly detection with stacked Transformer representations and 1D convolutional network. Eng. Appl. Artif. Intell..

[29] Gültekin Ö., Cinar E., Özkan K., Yazıcı A. (2022). Multisensory data fusion-based deep learning approach for fault diagnosis of an industrial autonomous transfer vehicle. Expert Syst. Appl..

[30] Li H., Boulanger P. (2022). Structural Anomalies Detection from Electrocardiogram (ECG) with Spectrogram and Handcrafted Features. Sensors.

[31] Zhou X., Xiong J., Zhang X., Liu X., Wei J. (2021). A radio anomaly detection algorithm based on modified generative adversarial network. IEEE Wirel. Commun. Lett..

[32] Chong S.H., Cho G.C., Hong E.S., Lee S.W. (2017). Numerical study of anomaly detection under rail track using a time-variant moving train load. Geomech. Eng..

[33] Kavalerov I., Li W., Czaja W., Chellappa R. (2020). 3-D Fourier scattering transform and classification of hyperspectral images. IEEE Trans. Geosci. Remote Sens..

[34] Khan A.S., Ahmad Z., Abdullah J., Ahmad F. (2021). A spectrogram image-based network anomaly detection system using deep convolutional neural network. IEEE Access.

[35] Haleem M.S., Castaldo R., Pagliara S.M., Petretta M., Salvatore M., Franzese M., Pecchia L. (2021). Time adaptive ECG driven cardiovascular disease detector. Biomed. Signal Process. Control.

[36] Sanakkayala D.C., Varadarajan V., Kumar N., Soni G., Kamat P., Kumar S., Patil S., Kotecha K. (2022). Explainable AI for Bearing Fault Prognosis Using Deep Learning Techniques. Micromachines.

[37] Liu C., Cichon A., Królczyk G., Li Z. (2021). Technology development and commercial applications of industrial fault diagnosis system: A review. Int. J. Adv. Manuf. Technol..

[38] Stankovic L., Ivanovic V., Petrovic Z. (1996). Unified approach to the noise analysis in the spectrogram and Wigner distribution. Ann. Telecommun..

[39] Friedlander B., Scharf L. (2023). On the structure of time-frequency spectrum estimators. IEEE Trans. Signal Process..

[40] Zhao H., Wang Y., Duan J., Huang C., Cao D., Tong Y., Xu B., Bai J., Tong J., Zhang Q. Multivariate time-series anomaly detection via graph attention network. Proceedings of the 2020 IEEE International Conference on Data Mining (ICDM).

[41] Entekhabi D., Njoku E.G., O’Neill P.E., Kellogg K.H., Crow W.T., Edelstein W.N., Entin J.K., Goodman S.D., Jackson T.J., Johnson J. (2010). The soil moisture active passive (SMAP) mission. Proc. IEEE.

[42] Srivastava N., Hinton G., Krizhevsky A., Sutskever I., Salakhutdinov R. (2014). Dropout: A simple way to prevent neural networks from overfitting. J. Mach. Learn. Res..

[43] Goldstein M., Uchida S. (2016). A comparative evaluation of unsupervised anomaly detection algorithms for multivariate data. PLoS ONE.

[44] Siffer A., Fouque P.A., Termier A., Largouet C. Anomaly detection in streams with extreme value theory. Proceedings of the 23rd ACM SIGKDD International Conference on Knowledge Discovery and Data Mining.

